# Viral Pandemics of the Last Four Decades: Pathophysiology, Health Impacts and Perspectives

**DOI:** 10.3390/ijerph17249411

**Published:** 2020-12-15

**Authors:** Shubhadeep Roychoudhury, Anandan Das, Pallav Sengupta, Sulagna Dutta, Shatabhisha Roychoudhury, Arun Paul Choudhury, A. B. Fuzayel Ahmed, Saumendra Bhattacharjee, Petr Slama

**Affiliations:** 1 Department of Life Science and Bioinformatics, Assam University, Silchar 788011, India; anandandas852@gmail.com; 2 Department of Physiology, Faculty of Medicine and Biomedical Sciences, MAHSA University, SP2, Bandar Saujana Putra, Jenjarom, Selangor 42610, Malaysia; pallav_cu@yahoo.com; 3 Department of Oral Biology and Biomedical Sciences, Faculty of Dentistry, MAHSA University, SP2, Bandar Saujana Putra, Jenjarom, Selangor 42610, Malaysia; sulagna_dutta11@yahoo.com; 4 Department of Microbiology, R. G. Kar Medical College and Hospital, Kolkata 700004, India; shatabhisha87@gmail.com; 5 Health Centre, Assam University, Silchar 788011, India; 6 Department of Obstetrics and Gynecology, Silchar Medical College and Hospital, Silchar 788014, India; drarunpc@gmail.com (A.P.C.); drfuzayel@rediffmail.com (A.B.F.A.); 7 Department of Pathology, Silchar Medical College and Hospital, Silchar 788014, India; drsaumbhatt@gmail.com; 8 Department of Animal Morphology, Physiology and Genetics, Faculty of AgriSciences, Mendel University in Brno, Zemedelska 1, 613 00 Brno, Czech Republic; petr.slama@mendelu.cz

**Keywords:** COVID-19, Ebola, HIV, influenza, SARS-CoV-2

## Abstract

The last four decades has witnessed some of the deadliest viral pandemics with far-reaching consequences. These include the Human Immunodeficiency Virus (HIV) (1981), Severe Acute Respiratory Syndrome Coronavirus (SARS-CoV) (2002), Influenza A virus subtype H1N1 (A/H1N1) (2009), Middle East Respiratory Syndrome Coronavirus (MERS-CoV) (2012), Ebola virus (2013) and the Severe Acute Respiratory Syndrome Coronavirus-2 (SARS-CoV-2) (2019-present). Age- and gender-based characterizations suggest that SARS-CoV-2 resembles SARS-CoV and MERS-CoV with regard to higher fatality rates in males, and in the older population with comorbidities. The invasion-mechanism of SARS-CoV-2 and SARS-CoV, involves binding of its spike protein with angiotensin-converting enzyme 2 (ACE2) receptors; MERS-CoV utilizes dipeptidyl peptidase 4 (DPP4), whereas H1N1 influenza is equipped with hemagglutinin protein. The viral infections-mediated immunomodulation, and progressive inflammatory state may affect the functions of several other organs. Although no effective commercial vaccine is available for any of the viruses, those against SARS-CoV-2 are being developed at an unprecedented speed. Until now, only Pfizer/BioNTech’s vaccine has received temporary authorization from the UK Medicines and Healthcare products Regulatory Agency. Given the frequent emergence of viral pandemics in the 21st century, proper understanding of their characteristics and modes of action are essential to address the immediate and long-term health consequences.

## 1. Introduction

Since time immemorial, mankind has been in a constant quest to overcome the threat of infectious diseases. The last 40 years has been no exception, as the world witnessed the emergence and reemergence of viral outbreaks, of which Human Immunodeficiency Virus (HIV) in 1981, Severe Acute Respiratory Syndrome Coronavirus (SARS-CoV) in 2002, H1N1 influenza virus in 2009, Middle East Respiratory Syndrome Coronavirus (MERS-CoV) in 2012, Ebola virus in 2013 and the Severe Acute Respiratory Syndrome Coronavirus-2 (SARS-CoV-2) in 2019-present, are noteworthy [1,2,3,4]. Emergence of infectious diseases directly impact human health outcomes paving the way to impaired sustainable development [5]. An estimated 34.3 million people worldwide were living with HIV/Acquired Immune Deficiency Syndrome (AIDS) by the end of 20th century [6]. The epidemic left millions of children orphaned, disrupted social life and eroded civil order and economic growth, too [7]. The consequences of SARS-CoV epidemic were fatal, affecting about 8098 people resulting in 774 deaths by February 2003 [8]. However, the outbreak identified a number of shortcomings in hospitals and community control systems in many of the affected regions [9]. The 2009 H1N1 influenza pandemic also had far-reaching consequences on global health, which impacted over 214 countries and caused over 18,449 deaths. With a persistent threat from earlier influenza epidemics, the scientific communities were much more prepared in mindset and infrastructure, which allowed for rapid and effective research on basic scientific aspects of the disease, with impacts on its control and lessons for future epidemics [10]. MERS-CoV, another coronavirus outbreak, had a very high case fatality rate among the recent pandemics, which is about 43% [11]. More recently during 2013–2016, the Ebola viral disease has been one of the largest of its kind in history which resulted in a huge public health menace with large-scale social and economic impact in the affected countries. This outbreak also presented opportunities for research that might help national and global healthcare systems to better prepare for future outbreaks [12].

As a new decade begins, the world engages in fighting to contain another novel virus of pandemic proportions, named SARS-CoV-2, which causes Coronavirus Disease 2019 or COVID-19. It represents one of the greatest public health emergencies in human history. The virus was first detected in December 2019 and isolated from several workers of the Wuhan seafood market in China who were suffering from pneumonia [13]. Shortly thereafter, the World Health Organization (WHO) declared it a global pandemic on 11 March 2020 [14]. SARS-CoV-2 is highly contagious and has currently spread across 220 countries and territories of the world [15]. As of 11 December 2020, SARS-CoV-2 infections have been confirmed in approximately 68.4 million people worldwide, of which about 45 million people have recovered from the virus and more than 1.5 million have succumbed to it. According to these statistics, the recovery and death rates of this disease are about 65.60% and 2.28%, respectively [15]. At present, supportive therapeutic strategies and mitigation measures to contain the virus remain the best weapons in the fight to control COVID-19. However, scientists around the world are striving to develop vaccines via accelerated processes in order to confer immunity to the public against the virus [1].

The present review aims to compare the available information pertaining to SARS-CoV-2 and other viruses from recent pandemics, in terms of their modes of actions and impact on human organs, which will facilitate interventions for their specific treatment and prevention.

## 2. Gender- and Age-Based Differences in the Susceptibility to Severe Acute Respiratory Syndrome Coronavirus-2 (SARS-CoV-2) Infection in Comparison with Other Viruses

Males and females of different age groups often vary in their general response to these viruses [16]. A statistical disparity in the prevalence of disease based on age and gender has been established in many viral outbreaks. During the SARS epidemic of 2002, patients below 25 years of age tended to present with mild to moderate illness, whereas those above 60 years of age had a mortality rate of more than 50% and presented with more severe symptoms [17]. In addition, epidemiological studies showed that males had higher fatality rates compared with that of females (21.9% versus 13.2%, respectively) [18]. Similarly, data from the MERS-CoV outbreak of 2012 showed that patients in the age groups of 45–59 years and above 60 years were more likely to be infected, suffer from more severe symptoms and have higher fatality rates compared with younger adults. Furthermore, the disease occurrence in males was higher than that in females, with fatality rates of 52% and 23%, respectively [19]. Another study also reported that among the confirmed cases of MERS-CoV, the male–female ratio was approximately 2:1 (67% male and 35% female) with highest prevalence of infected cases (41.2%) seen in the age group of 41–60 years [20]. Variations in disease prevalence among men and women may be attributed to the differences in cultural roles and gender norms that influence risk for contracting the disease. Women are more likely to employ themselves in essential services like healthcare and service industries compared to men [21]. However, men predominate in other sectors such as construction work and cleaning, security work, taxi services and low-skilled social care [22]. Although women are more proactive about their health when compared with men, they frequently receive less intensive diagnostic and treatment interventions, with women’s symptoms often being overlooked or assumed to be psychosomatic in many societies [23]. Viruses other than those in the family Coronaviridae include the H1N1 virus, which caused a pandemic in 2009 and primarily affected children and young adults of reproductive age, with the highest attack and hospitalization rates in individuals between the ages of 0 and 40 years [24]. The male-to-female morbidity ratio is more than one for this disease, suggesting that men are more susceptible to the H1N1 virus [25]. This may also be due to the differences in gender-based social stratifiers which influence the patterns of exposure to pathogens, vulnerability to illness and outcome of illness resulting in differences in incidence, duration, severity and fatality rates [26]. The likelihood of exposure to H1N1 virus was more in healthcare workers and people who work with children, professions predominantly employing women [24]. Differences in health-seeking behavior may also have significant impact on acquisition and manifestation of Influenza A. In most of the developing countries the quality of care received by women has mostly been compromised and has not been as good as that received by men [27]. The knowledge and awareness of the pandemic among the women has been less than that of men, which is a reflection of the unequal distribution of educational opportunities between men and women in such societies, with women being less privileged including the clinical aspects [28]. Furthermore, there has been disparity in hospitalization rates among minorities in high income countries in North America, too. A study confirmed significantly more hospitalization of H1N1 patients among ethnic minorities as compared to non-ethnic minorities. It was suspected that the non-ethnic minorities may have greater proportion of comorbidities, pregnancy or obesity—the known risk factors of pandemic H1N1 [29]. Another study conducted in the USA confirmed the race/ethnicity-related disparities in accessing healthcare for H1N1 patients. It was found that about 63% of Spanish-speaking Hispanics lacked regularity in healthcare provision, which was significantly different from Blacks, English-speaking Hispanics and Whites. Moreover, 43.6% of the Spanish-speaking Hispanics lacked money or insurance to get a flu shot in comparison to 23% of Whites, 23.3% of Blacks and 24.2% of English-speaking Hispanics [30]. Furthermore, the proportion of poor people with insurance (69.8%) was significantly lower than that of higher-income people (93.5%) [31]. Moreover, the outbreak of the Ebola virus in 2013 was most prevalent in adults of more than 30 years of age [32]. Although there are no prominent biological differences in gender-based susceptibility to Ebola infection, men and women differed in their exposure. Women are believed to be more emotionally attached and thus inclined to nurse their sick household members, too. This is also in accordance with the prevalent societal norms wherein women are considered as the primary caregivers to the diseased children and husbands. However, it is relatively less common for men to take such care of their family members during illness [33]. In this respect, higher death rates were seen in women, as their involvement in caring for the sick was higher [33]. In West Africa, a significant gender inequality has been noted in terms of susceptibility and healthcare access, and as a result women were rendered more vulnerable to Ebola infection. Socio-cultural barriers are believed to have denied women the access to proper health information and healthcare facilities [34,35]. The Ebola outbreak in Central and Eastern Africa also indicated the role of gender-related factors as key determinants of inequality in exposure and infection [36]. The grave ramifications of this are illustrated by estimated gender asymmetries in Ebola infection and fatalities [37]. There have also been evidences of racial discrimination of healthcare access of Ebola patients in the USA, and the minorities, the poor and the immigrants are not believed to receive the same care in the USA as their majority, affluent and native-born counterparts [38]. For instance, an Ebola patient travelling to the USA from Liberia was prematurely released from hospital as he lacked a health insurance just to be readmitted when his condition worsened [39,40]. During the HIV epidemic, the infection rate was high among younger populations of reproductive age (15–30 years), who accounted for 61.5% of the cases in East Africa [41]. In sub-Saharan Africa, about 60% of the individuals living with HIV/AIDS were women, particularly those in the age range of 15–24 years, indicating that their susceptibility to the disease was higher than that of men [42]. On the other hand, less than 25% of people living with HIV/AIDS in North Africa and the Middle East were women [43,44]. In the USA, women accounted for only 23% of new HIV infections [45]. In Nigeria, HIV-infected women have largely remained devoid of prevention and treatment services because of the prevalent socio-cultural norms, stereotypes and expectations. Young women of age range 15–24 years were affected twice as much as men of the same age. The inequalities remain evident even after death. A HIV-infected deceased man is buried with full ritual and rites but if it is a woman then the ceremony passes off without any elaborate funeral rites [46]. In the USA, racial and ethnic disparities were also witnessed during the HIV epidemic, especially in the Western societies where African-Americans are less likely to have an infectious disease specialist as a regular source of care in comparison with White patients. Natives of Alaska, American-Indian, Asian, Pacific Islander or mixed racial background have also been less likely to have an infectious disease specialist than the Whites [47]. According to the US Centers for Disease Control and Prevention (CDC), African-American and Hispanics accounted for 42% and 27% of the HIV diagnoses. Certain subpopulations within ethnic and racial minority groups such as Black African-American gay, bisexual and other men who have sex with men were more affected by HIV than any other group in the USA [48].

Clinical and epidemiological data suggest that the prognosis of COVID-19 is worse in patients aged above 60 years than those who are younger than 60. Patients under 60 years tend to have less severe symptoms and higher recovery rates than older patients [49]. Studies have shown that older patients (more than 60 years of age) suffering from COVID-19 have an increased risk of death compared with younger adult patients (aged less than 60 years) [50]. The risk of disease occurrence and death increases substantially in older patients who suffer from comorbidities, such as diabetes, hypertension and pulmonary and respiratory diseases. Epidemiologically, men are at greater risk of infection and severe COVID-19 outcomes than women [51]. There are roughly similar numbers of confirmed cases between men and women [52], however, the sex bias in COVID-19 fatality has been confirmed. Reports from China, South Korea, the USA and several other European countries have indicated higher fatality rates in male patients in comparison with females [53,54,55]. Female patients are also in less demand of intensive care and are also significantly less likely to develop the severe form of the disease [56,57]. It is worth mentioning that worse outcomes and higher deaths in men as compared with women have so far been independent of age [57]. Furthermore, male patients with comorbidities have a higher risk of getting critically ill compared with men without comorbidities; whereas there is no such association in women [58]. Symptoms such as cough and fever are experienced more by men, too [59]. While men and women have an equal prevalence of disease occurrence, the death rate is about 2.4 times higher in males than in females, and the age data of deceased patients have revealed comparable trends between men and women [57].

Thus, a comparison of the age- and gender-based characteristics between SARS-CoV-2 and other viruses suggests that the novel virus most resembles SARS-CoV and MERS-CoV in this regard.

## 3. Mechanism of Host Cell Invasion of SARS-CoV-2 in Comparison with Other Viruses

Viruses possess unique strategies to invade host cells. Although individual viral entities have specific variations in their mechanisms of host cell invasion, the overall process can be somewhat generalized. To gain access to the interior of the cell, enveloped viruses fuse directly with the cell plasma membrane, whereas other viruses need to be endocytosed by the host cells. The introduction of viral genetic material to the host cell soon leads to intracellular disruptions [60].

Enveloped viruses contain fusion proteins on their surfaces that interact with cell–surface receptor proteins [60]. The process of fusion comprises two major steps. First, the monolayers of the virus and the cell merge in a process called hemifusion, which is followed by the collapse of unmerged monolayers into each other to create a single bilayer, called the hemifusion diaphragm. In the second step, the single bilayer is disrupted by a pore created by fusion proteins, through which the viral genome gains entry to the interior of the cell [61]. Once inside the cell, the virus hijacks the cellular machinery to produce virally encoded proteins that replicate the genetic material of the virus [60].

H1N1 influenza A virus, which is equipped with the hemagglutinin protein, initiates the infection process in the respiratory tract. The virus attaches to receptor glycoconjugates of unknown identity with linearly placed terminal α-2,3-linked sialic acid residues [62,63]. Hemagglutinin-mediated binding to the receptor triggers endocytosis of the virion, which can occur in a clathrin-dependent manner or through macropinocytosis [64,65]. This is followed by the opening of the matrix 2 protein ion channel, which acidifies the inside of viral particles and subsequently releases viral RNA [65,66]. The invasion mechanism of the Ebola virus has been partially explored; it infects a variety of cellular targets, such as endothelial cells, fibroblasts, hepatocytes and adrenal cortical cells [67]. The virus has surface glycoproteins that attach to various cell surface receptors in the C-type lectin family of proteins. The family includes asialoglycoprotein receptors, dendritic cell-specific intercellular adhesion molecule (ICAM)-3-grabbing non-integrin, human macrophage galactose, acetylgalactosamine-specific C-type lectin and lymph node sinusoidal endothelial cell C-type lectin, which have all been shown to interact with Ebola virus surface glycoprotein [68]. Following attachment, virus particles are taken up by various endocytic pathways, such as clathrin-dependent and caveolin-dependent pathways and macropinocytosis [68,69]. Recent studies have shown that the endocytic pathway of Ebola virus entry is dependent on the enzyme cathepsin, which cleaves the viral glycoprotein in acidic conditions, thus facilitating the internalization of the viral genome [70,71]. HIV invades the male and female genital tracts through the mucosal epithelial surface. Glycoprotein 120 (gp120) on the surface of HIV interacts with two surface glycosphingolipids—sulfated lactosylceramide and galactosyl ceramide on the vaginal and ectocervical epithelium, respectively—to initiate transcytosis [72,73,74]. Once within the epithelium, HIV encounters and binds directly to CD4+ T cells and dendritic cells [75]. Gp120 and gp41 on the surface of the virus attach to CD4 and chemokine coreceptors, such as C-C chemokine receptor type 5 (CCR5) or C-X-C chemokine receptor type 4 (CXCR4) of the leukocyte, which is followed by endocytosis of the virus, although other non-endocytic pathways of entry also exist [76]. Membrane labeling studies have shown that the viral envelope fuses with the endocytic compartment, releasing the viral genome and enzymes into the cytosol [77].

SARS-CoV and MERS-CoV belong to the Coronaviridae family and β-Coronavirus subtype, and thus, they have fairly similar patterns of host cell invasion. As mentioned earlier, the first step of virus entry is the interaction between viral spike proteins and receptors on the cell. The receptor-binding domain of these coronaviruses resides in the C-terminus of the spike protein sub-segment S1 [78]. Different coronaviruses use different cellular receptors for their entry: for example, as cell surface receptor proteins, SARS-CoV uses angiotensin-converting enzyme 2 (ACE2), which is expressed in vascular endothelial cells, renal tubular epithelium and various other organs, while MERS-CoV utilizes dipeptidyl peptidase 4 (DPP4), which is expressed on the surface of most cell types [79,80,81]. Acid-dependent proteolytic cleavage of viral spike proteins leads to the fusion of viral and host cell membranes in the acidic environment of the endosome. Ultimately, an antiparallel six-helix bundle is formed from the cleaved spike protein, which allows for the mixing of viral and cellular membranes, leading to the release of the viral genome into the cytosol [82,83]. 

The invasion mechanism of SARS-CoV-2, similar to SARS-CoV, is initiated when its spike protein comes into contact with the ACE2 receptor on the cell surface of the target organ [84]. This is followed by the fusion of the viral membrane and the host cell [85]. After fusion, a conformational change in the viral spike protein is initiated by type II transmembrane serine protease on the cell surface, which allows the virus to enter the cell [86]. The highest ACE2 expression has been detected in nasal epithelial cells and ciliated secretory cells of the respiratory tract, which is the prime reason that these tissues are the primary target of this virus [87]. Recently, high-sensitivity RNA in situ mapping revealed a striking gradient of SARS-CoV-2 infectivity along the nasal pulmonary epithelial tissue, with a relatively high rate of infection in the proximal portion of the lungs in comparison with the distal portion. Such variations are the result of the higher nasal ACE2 expression levels in the bronchial pathway in the proximal portion and their progressive decline towards the distal portion [88]. ACE2 has also been detected in the stomach, small intestine, colon, skin, lymph node, thymus, bone marrow, brain, spleen, liver, kidney and reproductive tract; in fact, it is expressed in the endothelial and smooth muscle cells of virtually all organs, which is suggestive of the fact that SARS-CoV-2 not only invades the respiratory system but also poses potential threats to digestive, urogenital, circulatory, central nervous and reproductive systems [89]. Upregulation of ACE2 following inflammation may increase the susceptibility of several tissues to further damage that may lead to multiple organ failure in severe cases of SARS-CoV-2 infection [90]. Once inside the cell, the virus releases its genetic material and starts the process of viral replication, which is followed by the assembly of numerous viral particles and their release from the cell [91]. The process of replication in coronaviruses is unique among RNA viruses in the sense that the viral RNA synthesizes replicase and other non-structural proteins with the help of host ribosomes and ultimately forms the replicase–transcriptase complex. The process of transcription produces genomic and sub-genomic RNA that further undergoes translation to synthesize viral structural proteins [83,92].

From this comparative analysis of invasion mechanisms of SARS-CoV-2 and other viruses, SARS-CoV-2 clearly shares similarities with SARS-CoV in the overall pattern of invasion and receptor specificity. Furthermore, evidence of organ-to-organ transmission of SARS-CoV-2 based on the presence or absence of the ACE2 receptor has also been reported. Therefore, in addition to the respiratory system, the virus may infect other organs, including the cardiovascular, gastrointestinal, nervous, renal and reproductive systems. 

## 4. Effects of SARS-CoV-2 and Other Viruses on Major Physiological Processes

**Figure 1:**
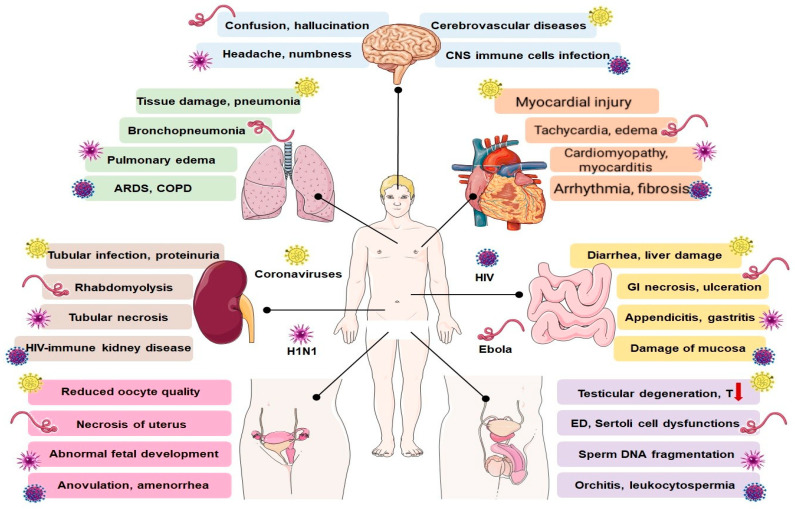
Effects of different viruses on various physiological processes of the human body. The illustration summarizes the health impacts of viruses on different organ systems. ARDS—acute respiratory distress syndrome; COPD—chronic obstructive pulmonary disease; ED—erectile dysfunction; GI—gastrointestinal; T—testosterone.

Numerous viral entities are known to affect different human organ systems and hamper their proper functioning. SARS-CoV-2 is included in the long list of viruses that affect multiple physiological functions in humans. Although the respiratory system is the primary site affected, infection with this virus has also proved to be a major threat to other vital organs, especially to those with high expression of the ACE2 receptor [93]. Figure 1 depicts the susceptible systems, which include the cardiovascular, gastrointestinal, renal, central nervous and reproductive systems [93]. In this segment, the impacts of SARS-CoV-2 and other relevant viruses on major physiological processes are discussed.

### 4.1. Respiratory System

H1N1 influenza virus causes acute respiratory disease, which is a consequence of an overactive inflammatory response and virus-induced cytokine dysregulation. Viral invasion results in excessive production of numerous pro-inflammatory cytokines, which leads to the development of clinical conditions such as pulmonary edema, acute bronchopneumonia, alveolar hemorrhage and Acute Respiratory Distress Syndrome (ARDS) [94]. Patients suffering from Ebola virus disease are also at risk of developing respiratory problems. The pulmonary pathophysiological effects of Ebola virus infection include tachypnea with increased oxygen requirements and vascular leakage, which leads to subsequent pulmonary edema [95]. The Ebola virus severely affects the respiratory system prior to its manifestation. Its mechanism of action is uncertain, but it may be attributable to direct viral invasion and replication and the subsequent unregulated release of pro-inflammatory mediators [96]. HIV-infected persons are also at risk of suffering from chronic pulmonary disease and other respiratory dysfunctions. The clinically relevant pulmonary conditions of patients suffering from AIDS are chronic obstructive pulmonary conditions, gas exchange abnormalities, asthma and cardiopulmonary diseases. The underlying mechanism includes microbial translocation and resultant inflammation, caused by macrophage activation, endothelial dysfunction, oxidative stress and changes in the host microbiome [97]. SARS-CoV predominantly affects the respiratory tract of the patient. Some of the major effects include extensive alveolar collapse, fluid-filled and desquamated alveoli, alveolar epithelial hyperplasia and damaged bronchial epithelial cells. These effects lead to the development of adult respiratory distress syndrome, with minimal infiltration of inflammatory mediators [98]. Even in cases of MERS-CoV-infected patients, acute respiratory illness with mild to severe respiratory symptoms is common. Chest X-rays have shown lung ground-glass opacities, pleural thickening and fibrosis with moderate frequency in patients. Severe MERS-CoV infection ultimately progresses to acute respiratory distress syndrome [99].

CoVs have become the major pathogens of emerging respiratory disease outbreaks, and SARS-CoV-2 is not an exception to this. The majority of symptomatic patients develop mild flu-like symptoms such as cough, fever and difficulty in breathing, while others may develop severe lung injury together with ARDS [100]. SARS-CoV-2 induces pneumonia in patients with mild to moderate severity. The viral invasion is followed by unrestricted inflammatory responses and ‘cytokine storms’ that affect the host cells. The result may be extensive tissue damage with dysregulation of coagulation and microvascular pulmonary thrombosis [101]. Further worsening of the situation may lead to ARDS with different degrees of severity, with hypoxia being a distinguishing symptom [2].

### 4.2. Cardiovascular System

The risk of cardiovascular diseases may increase during viral infection [102]. H1N1 influenza virus has a wide range of effects on the heart and the circulatory system. Endomyocardial biopsies have confirmed the presence of active inflammation and necrosis in myocardial tissue, which later gives rise to dilated cardiomyopathy and myocarditis [103,104]. Furthermore, the risk of acute myocardial infarction, chronic ischemic heart disease, stroke and sudden cardiac death markedly increases in H1N1-infected patients [105]. Patients infected with the Ebola virus are also under constant threat of cardiovascular and pulmonary diseases. With the onset of fever, patients may develop tachycardia with progressive hypotension [106]. Increased inflammation due to virus-induced cytokine circulation may result in decreased systemic vascular resistance, decreased ventricular inotropy and decreased contractility of the heart [95]. Recent studies have confirmed the increased risk of cardiovascular diseases in HIV-infected patients. As a result of chronic inflammation, infected patients remain at high risk of left ventricular systolic and diastolic dysfunction, myocardial fibrosis, regional myocardial dysfunction, coronary artery disease, arrhythmias such as atrial fibrillation and sudden cardiac death [107]. In SARS-CoV-infected patients, cardiovascular complications are common, among which hypotension and tachycardia are the most common clinical manifestations. Other clinical complications, such as brachycardia, cardiomegaly and cardiac arrhythmia, are of rare occurrence in these patients [108]. Chronic cardiac disease frequently occurs in MERS-CoV-infected patients as well, resulting in an increased rate of mortality in infected patients [109].

Clinical data show that patients infected with SARS-CoV-2 have a higher tendency to develop circulatory symptoms such as palpitations, chest tightness and shortness of breath as initial symptoms [110]. In some cases, patients experience a sudden progressive decline in heart rate, during which heart sounds may become clinically undetectable. The high risk of cardiovascular complications in SARS-CoV-2-infected patients may be due to the increased release of cytokines in the body, which ultimately leads to inflammatory responses [89]. 

### 4.3. Gastrointestinal System

Viral diseases also have the potential to disrupt the normal functioning of the gastrointestinal (GI) system. H1N1 influenza virus can induce severe GI complications such as acute appendicitis, abdominal pain and hemorrhagic gastritis in severe cases, especially in children [111,112]. In the early stages of Ebola infection, patients experience gastrointestinal necrosis and hemorrhage, accompanied by ulcerations in the GI tract. Multiple severe implications can arise in the subsequent stages of Ebola infection, which include serosal bleeding, congestion in the GI junction, focal erosions and thrombosis of submucosa and lamina propria. Ebola virus infection also leads to necrosis of the gastric-associated lymphocyte tissue (GALT), which facilitates the introduction of other pathogenic bacteria due to compromised immune functions [113]. HIV-infected patients also exhibit GI disorder symptoms, of which diarrhea is the most common. Over the course of infection, extensive infiltration of virus-laden lymphocytes damages the protective mucosal barrier, which increases the probability of attack by opportunistic pathogens. Histological sampling of the small intestine and colon of HIV-infected patients have revealed both structural and immunological abnormalities, which include villous atrophy, crypt hyperplasia, epithelial hypoproliferation and CD4+ depletion within lamina propria [114]. The effect of SARS-CoV on the GI system is also well documented. In the early phase of infection, typical symptoms include diarrhea, nausea, vomiting and abdominal pain. Increased aminotransferase levels, which indicate liver damage, manifest in later stages of infection. Pathological evaluations have revealed regional hemorrhages, vascular congestion and lymphocytic infiltration in the gut wall [115]. One-third of MERS-COV-infected patients report GI symptoms, which include abdominal pain, nausea, vomiting and diarrhea [116]. The primary intestinal epithelial cells, small intestine and intestinal organoids show tissue degeneration and inflammation due to increased viral load [117].

Available reports suggest that SARS-CoV-2 infection is also associated with GI dysfunctions; however, they are less severe than those reported in earlier coronavirus outbreaks, namely, SARS and MERS-CoV. Nonetheless, a significant proportion of infected patients present GI symptoms such as diarrhea, nausea, vomiting and abdominal pain, with mild to moderate elevations in levels of liver enzymes [118].

### 4.4. Nervous System

All of these viruses are able to invade the nervous system. Patients suffering from H1N1 influenza virus have mild to severe neurologic complications. In most cases, the complaints are mild and include headache, numbness and paresthesia, vertigo, drowsiness and weakness. Severe neurological complications include seizures, acute inflammatory demyelinating polyneuropathy, acute disseminated encephalomyelitis and alterations in the level of consciousness, ranging from lethargy to coma [119,120]. In Ebola virus-infected patients, neurologic symptoms are infrequent, with headache being the most common symptom. Altered mental status, mild confusion and hallucination may also occur, accompanied by electrolyte imbalance, shock and coma in severe cases [121]. In recent outbreaks, meningitis and encephalitis have also been reported, which may be accompanied by short-term memory loss, hypomania, hyperphagia and insomnia [122]. HIV is also capable of affecting the central nervous system (CNS) in two ways: primary HIV CNS disease, for which the virus is both necessary and sufficient, and secondary CNS disease, in which opportunistic pathogens take advantage of the compromised immune system. HIV causes neuronal damage by infecting immune cells of the CNS. Severe symptoms of HIV-induced neurological disorders range from asymptomatic neurocognitive impairment to HIV-associated dementia [123]. In the long term, HIV infection also affects motor functions and coordinated regulations of movements by the CNS [123]. There are reported cases of SARS-CoV infection in which patients developed neurologic symptoms such as seizures, myopathy and rhabdomyolysis [124]. In some cases, acute cerebrovascular diseases have been reported with the evident presence of viral RNA in both cerebrospinal fluid (CSF) and brain tissue [124,125]. Neurologic disorders were also reported during the MERS-CoV outbreak: such patients experienced neuropathy, delirium and acute cerebrovascular disease [126]. Other reports have documented seizures and confusion in infected patients, but the presence of MERS-CoV viral particles in the CSF has not been established, in contrast to SARS-CoV [127].

There is evidence to support the potential effects of coronavirus infection on the human nervous system. However, it is difficult to ascertain the exact neurological complications associated with the overall pathophysiology. Mechanistically, it is well established that SARS-CoV-2 interacts with the ACE2 receptor protein in the capillary endothelium and causes blood–brain barrier destruction, ultimately promoting virus entry into the CNS. Although not definitively demonstrated, another possible mechanism may involve the release of excessive levels of various pro-inflammatory factors that ultimately promote neuroinflammation following the viral infection [128]. Typical neurological symptoms of infected patients include headache, epilepsy and confusion, and some patients have a high risk of intracranial hemorrhage. Similar to SARS-CoV, SARS-CoV-2 RNA in the CSF and brain tissues has been confirmed in COVID-19 patients. SARS-CoV-2 viruses also have the potential to migrate to sensory and motor nerve endings and even the brainstem, which controls the vital functions of the body [89].

### 4.5. Renal System

Renal complications are also common in the pathogenesis of most of these viral infections. H1N1 influenza virus is known to infect the kidneys, and histological examination has confirmed acute tubular necrosis, myoglobin pigmentation and disseminated intravascular coagulation. Patients with severe infection are likely to develop acute kidney injury, rhabdomyolysis, hemolytic uremia syndrome, acute glomerulonephritis, Good pasture’s syndrome and acute tubulointerstitial nephritis [129]. Kidney injury is the most common renal complication in Ebola virus-infected patients. The leading causes of kidney injury range from volume depletion due to diarrhea to increased vascular permeability, bacterial infection, cytokine storm and rhabdomyolysis [130]. HIV-infected patients are also at higher risk of developing kidney disease than non-infected individuals. In an infected individual, kidney disease manifests in a number of ways, including acute kidney injury, HIV-associated kidney disease, comorbid chronic kidney disease and treatment-related kidney toxicity. The first described renal dysfunction in HIV-infected patients was HIV-associated neuropathy, which is commonly observed in patients who are newly diagnosed with late-stage infection. In association with this, the spectrum of HIV-associated kidney disease includes HIV immune complex kidney disease and, less commonly, thrombotic microangiopathy [131]. In situ hybridization techniques have confirmed the presence of SARS-CoV particles in the epithelial cells of renal distal tubules and in the cytoplasm of the distal tubular epithelium [98]. The development of acute renal failure in infected patients is common but is often associated with indirect causes such as pre-renal factors, hypotension, rhabdomyolysis and previous comorbidities such as diabetes and old age [132]. Patients infected with MERS-CoV are at risk of developing progressive renal function impairment. Early in the outbreak, MERS-CoV-infected patients with severe pneumonia and acute respiratory distress syndrome were observed to have had acute kidney injury thereafter, which is suspected to be the result of factors such as the virus itself, associated systemic inflammation and hypotension. Half of infected patients are also likely to suffer from proteinuria [133].

In contrast to earlier studies on SARS- and MERS-CoV-infected patients, recent studies have shown that the human kidney is a potential site for SARS-CoV-2 infection due to the presence of ACE2 surface receptors in the kidneys. Because of the increased affinity of SARS-CoV-2 towards ACE2, there is an increased viral load in the kidney, specifically in the proximal tubular epithelium and in podocytes [134]. The most frequent kidney dysfunction in infected patients is mild to moderate proteinuria, which is partially a consequence of direct podocyte infection with potential rennin–angiotensin–aldosterone system alterations, which together affect the glomerular filtration barrier and result in the increased filtration of plasmatic proteins [135].

### 4.6. Reproductive System

Viral infections may affect both male and female reproductive functions either via direct invasion or through secondary inflammatory pathways. Strong evidence confirms the effects of H1N1 on human sperm quality. Studies have shown that influenza can have latent effects on sperm DNA integrity and may result in the transient release of abnormal sperm [136], and it is even associated with the risk of infertility [137]. The H1N1 virus reportedly has pronounced impacts on pregnant women and fetal development. The risk of morbidity from seasonal influenza is higher in pregnant females than in the general population [138]. In the fetus, complications due to the direct effects of the virus in the mother include fetal tachycardia and febrile morbidity, whereas indirect effects due to hyperthermia include neural tube defects and other congenital anomalies, such as cerebral palsy, neonatal seizures, newborn encephalopathy and even death [139].

The transmissibility of the Ebola virus through sexual contact is well established, although its effects on the human reproductive system are not well documented. A study in a macaque model indicated that Ebola virus replication may occur predominantly within the mesenchymal or supporting stromal cells of the reproductive tract [140]. However, the presence of Ebola virus around ovarian follicles in thecal mesenchymal cells has been positively associated with inflammation and necrosis in the uterus in a guinea pig model [141]. Furthermore, maternal and fetal mortality may considerably increase among pregnant women with Ebola infection [142]. Additionally, this virus is transmissible from an infected mother to the child via breastfeeding [143].

In cases of HIV, infected males suffer from impaired semen quality, including semen volume, sperm motility, concentration and morphology [144]. HIV-infected males are also likely to develop orchitis, hypogonadism and leukocytospermia, and patients in advanced stages of the disease may suffer from erectile and ejaculatory dysfunctions [145]. Women infected with HIV are more likely to have protracted anovulation and amenorrhea [146]. Secondary infection due to the disease may also lead to infertility [147]. Furthermore, the chances of pregnancy loss are more common among HIV-infected women as compared with healthy women [148].

Data regarding pregnancy-related complications of MERS-CoV infection are limited, and to date, only two such cases are known. The first reported case was a stillbirth at 5 months of gestation in a Jordanian woman with MERS-CoV infection [149]. The second case was from the United Arab Emirates: a woman with MERS-CoV infection died during the third trimester of pregnancy after giving birth to a healthy baby with no signs of MERS-CoV infection [150].

Data on the infection potential of coronaviruses in the human reproductive system can be traced back to the SARS-CoV epidemic of 2002. SARS-CoV-2 has been speculated to act in a similar manner when it affects reproductive functions. The male reproductive system expresses higher levels of the ACE2 receptor compared with the female reproductive system, which may explain the increased vulnerability of male reproductive functions to the effects of SARS-CoV infection relative to female reproductive functions [151]. Sperm cells, Leydig cells and Sertoli cells are known to express high levels of the ACE2 receptor, but some studies have reported that SARS-CoV and SARS-CoV-2 could not be detected in the semen sample of patients [152,153]. However, there are contradictions in this regard because a recent study confirmed the presence of SARS-CoV-2 virus particles in semen [154]. The virus may reach the semen via the impaired blood–testis barrier in the presence of systemic/local inflammation [154]. Studies have shown that the level of serum luteinizing hormone (LH) is significantly increased in SARS-infected patients. However, infected patients have markedly decreased serum testosterone levels, along with a significant reduction in the ratio of testosterone to LH and the ratio of follicle-stimulating hormone (FSH) to LH, which is suggestive of the fact that SARS-CoV directly affects the testicular tissue rather than affecting the hypothalamus–pituitary–gonadal (HPG) axis [155,156]. 

Other indirect effects, such as damage to germ cells and testicular dysfunctions due to a persistent rise in body temperature in response to the virus infection, have also been reported. Hyperplasia of Leydig cells in infected patients has been observed in some cases. Leydig cell dysfunction, reduced testosterone production, destruction of seminiferous epithelium and damage to the blood–testis barrier are some of the notable effects of possible inflammatory responses arising from SARS-CoV infection [151].

Studies have shown that coronaviruses in previous outbreaks, such as SARS-CoV, are associated with orchitis, which may lead to the disruption of spermatogenesis and germ cell apoptosis, thereby affecting semen quality [151]. Immunohistochemistry has confirmed immunoglobulin G (IgG) deposition in testicular tissues, although viral genomic material has not been detected in testicular tissue or seminal plasma. This is an indication that inflammatory and immunologic reactions may play a vital role in virus-mediated testicular damage and the induction of oxidative stress [157]. Moreover, stress and anxiety are potent modulators of oxidative stress in the body, and abundant data support the existence of a link between oxidative stress and high-anxiety-related behavior, such as depression, stress and post-traumatic stress disorder; however, the underlying cause–effect relationship is yet to be established [158,159]. Moreover, similar to SARS-CoV, SARS-CoV-2 is postulated to adopt the stress-evasion strategy via amino acid sequences that mimicthe host adrenocorticotropic hormone (ACTH) and thus trigger antibodies against the host’s self ACTH molecules. This mechanism will suppress the stress-induced increase in host cortisol levels that would otherwise aid in combating stress and inflammation [160,161]. Thus, unrestricted inflammation continues to adversely affect the organs. The resultant oxidative damage at micro-levels leads to membrane lipid peroxidation and sperm DNA fragmentation, which negatively impacts testicular functions such as spermatogenesis and spermiogenesis. Furthermore, sperm count and seminal volume are lowered, which may adversely affect reproductive outcomes and ultimately lead to infertility in males [162]. SARS-CoV-infected women are also likely to have disrupted sexual function due to stress, and this may negatively impact the oocyte quality, menstruation and fecundity [163]. 

Coronaviruses may also affect women’s health and well-being, particularly those who are pregnant. During the previous SARS-CoV pandemic, viral infection was associated with adverse pregnancy outcomes, including miscarriage, premature delivery and respiratory distress syndrome in newborn infants [164]. In the recent COVID-19 pandemic, SARS-CoV-2 has not yet been shown to cause any significant damage to the female reproductive system. However, it is hypothesized that SARS-CoV-2 infection may affect female fertility by decreasing ovarian function and oocyte quality and increasing the chances of miscarriage [165]. It has also been posited that SARS-CoV-2-infected women are more vulnerable to developing pneumonia [166], which may further give rise to other complications, such as rupture of the membrane, preterm labor, intrauterine fetal death, intrauterine growth restrictions and neonatal death in pregnant women [167]. A recent study on the obstetric and perinatal aspects of SARS-CoV-2 reported premature deliveries in some cases, but no fetal death, neonatal death or neonatal asphyxia was reported [168]. Premature labor was further confirmed in some COVID-19 patients without any notable vertical transmission [169].

In the male, physiological stress may lead to decreased sperm quality and enhanced sexual dysfunction [170]. This may be accompanied by inhibitory effects on the HPG axis, thereby affecting testosterone levels, which, in turn, may induce changes in Sertoli cells and the blood–testis barrier, leading to the arrest of spermatogenesis [171]. 

Table 1 summarizes the comparative aspects of the pathophysiologies of the aforementioned viruses, along with the respective treatment strategies. The table also highlights the epidemiological characteristics and the immunological responses elicited by the viruses.

## 5. Outlooks on Vaccine Development for SARS-CoV-2 in Reference to SARS and Middle East Respiratory Syndrome Coronavirus (MERS-CoV)

As previously discussed, before the SARS-CoV-2 pandemic, the world suffered from two deadly epidemics of coronaviruses in the past, viz. SARS-CoV and MERS-CoV in 2002 and 2012, respectively. However, no effective commercial vaccine has been developed for either of these coronaviruses for different reasons. Nevertheless, the past efforts and current knowledge of vaccine development for these viruses may prove to be valuable for the development of an effective vaccine for COVID-19 [199].

After the outbreak of the 2002 SARS-CoV epidemic, laboratories around the world started to conduct tests and clinical trials for the development of its vaccine. Vaccines based on the live-attenuated virus, live-attenuated recombinant virus, recombinant modified vaccinia virus Ankara, recombinant non-replicating adenovirus, virus-like particles and a combination of DNA, recombinant viral vector and viral peptides were used in the development of a SARS-CoV vaccine, which reached pre-clinical trials [200,201]. Only a few approaches reached the clinical phase I trial stage. These include inactivated SARS-CoV, DNA-based vaccines and soluble proteins. The DNA-based vaccines and soluble proteins targeted the spike proteins of the virus or its fragments. The former induced neutralizing antibodies and T-cell responses after 2–3 weeks in human trials, while the latter induced neutralizing antibodies in rabbits and stopped viral replication in mice. The inactivated SARS-CoV virus targeted all of the viral proteins and caused the significant induction of neutralizing antibodies in humans after two immunizations with no severe adverse effects [200,201,202,203]. Similarly, during the MERS-CoV epidemic in 2012, several potential vaccines were tested, but only a DNA-based vaccine was tested in the clinical phase I trial stage. This vaccine type targeted the viral spike proteins and its subunits and induced neutralizing antibodies and T-cell responses after three doses with moderate and mild side-effects. Other vaccine types, such as physically inactivated MERS-CoV virus, soluble proteins, nanoparticles and combination vaccines, reached the pre-clinical trial stage [204].

The development of an effective vaccine for SARS and MERS-CoV was decelerated owing to a lack of suitable animal models for testing. Although the animals developed immunological responses, they show limited viral replication and clinical manifestation of the disease [205]. This problem was addressed by developing transgenic animals that were rendered more permissive to coronavirus infection. For example, transgenic mice were created to express ACE2—the human cell receptor of SARS-CoV—which enhanced the infection sensitivity and facilitated the evaluation of protection from a lethal dose of the virus [206]. Transgenic mouse models that have the potential to express the human cell receptor of SARS-CoV-2 are now commercially available, which could be beneficial in the process of vaccine development. Ideally, a vaccine provides long-term protection. In SARS-CoV, although high titers of neutralizing antibodies were detected after passive immunization approaches, these antibodies could only be tracked for about 24 h, whereas memory T-cells were detected even six years after infection [207]. In the case of MERS-CoV, neutralizing antibodies persisted for about three years, whereas memory T-cell was detected two years after infection [208,209]. The question of whether a certain vaccination regimen can induce long-term protection has been explored in a few animal experiments. In the case of SARS-CoV, viral vectors and protein-based vaccines produced a certain level of protection from infection in mice after 4–12 months of vaccination [210]. Protein-based vaccines and a combination of DNA and protein-based vaccines used against MERS-CoV have shown some degree of long-term protection in mice and macaques [209].

As of 8 December 2020, 162 candidates for SARS-CoV-2 were in the pre-clinical trial phase, and 52 candidates in the clinical trial phase, of which 10 were in phase III clinical trial phase, 16 in phase II and the remaining 22 in phase I. Phase III clinical trial candidate vaccines are non-replicating viral vector, inactivated, RNA and protein subunit vaccines. The candidates in phase II clinical trials are non-replicating viral vector, inactivated, DNA, RNA and protein subunit vaccines. The vaccine platforms of phase I candidates include RNA, replicating viral vector, virus-like particles and protein subunits [210]. 

Vaccines against COVID-19 are being developed at an unprecedented speed, and the phase III clinical trials have been recruiting thousands of volunteers. For example, Pfizer/BioNTech already recruited 43,538 participants as on 27 July 2020 whereas Moderna’s mRNA-1273 had enrolled 30,000 volunteers as on 22 October, 2020 [211,212]. These vaccine development projects are using state of the art technologies for ensuring the safety and efficacy which may also seek to modernize other vaccines that are already in use globally. On 11 August 2020, the Russian Ministry of Health registered the first COVID-19 vaccine, developed by The Gamaleya National Center for Epidemiology and Microbiology, which is currently undergoing a phase III clinical trial by the name of ‘Sputnik V’. It is an adenovirus vector-based vaccine, and it is claimed to elicit a strong immune response in the body. The first batch of vaccine has already been released for distribution to the public, and further large-scale regional circulation is planned in the near future [213]. Moderna Therapeutics has created an mRNA vaccine, ‘mRNA-1273′, which is currently undergoing phase III clinical trials; mRNA-1273 can mimic many aspects of the real SARS-CoV-2 virus and can induce an effective immune response [213]. Pfizer and BioNTech have collaboratively developed an mRNA vaccine called ‘BNT162b2′, and it is currently undergoing phase III clinical trials. BNT162b2 has produced positive results in phases I and II, including the production of antibodies and T-cell responses specific to SARS-CoV-2 structural proteins [213]. On 2 December 2020 Pfizer and BioNTech’s vaccine received temporary authorization from the UK Medicines and Healthcare products Regulatory Agency, and on 2 December 2020 at 6.31 am local time in London, UK, Margaret Keenan, a 90-year-old woman, became the first person in the world to receive a clinically approved vaccine 334 days after the first reported COVID-19 death in China [214]. The University of Oxford and AstraZeneca, in a collaborative venture, have developed a non-replicating viral vector designated ‘ChAdOx1 nCoV-19′, which is also under a phase III clinical trial [213]. Three Chinese companies, Sinovac, Sinopharm and Cansino Biologics, have independently developed vaccines that are currently under phase III trials and have shown positive results along with mild symptoms in the recipient [213]. Novavax Incorporative, an American company has been testing a protein subunit vaccine which is currently undergoing phase III clinical trial, has elicited robust antibody response during phase I [215]. China-based Anhui Zhifei Longcom Biopharmaceutical Company in collaboration with the Institute of Microbiology, Chinese Academy of Sciences has developed another protein subunit vaccine which is currently being tested under a phase III clinical trial due to its efficacy in eliciting a potent immune response during previous phases [216,217]. Table 2 summarizes key information regarding the seven aforementioned vaccines currently undergoing phase III clinical trials.

## 6. Lessons Learned from the COVID-19 Pandemic and Other Viral Epidemics

The emergence of pandemics has shown that humans are not infallible, and the global population needs to be prepared for outbreaks to act appropriately and restore both the health and economy of affected nations [223]. The past SARS outbreak in 2003, the following H1N1 pandemic, and the Ebola outbreak each caused more than US $10 billion in economic damage. Economic impacts of the current COVID-19 pandemic have already been devastating, and the exact estimate of the loss will be available once the SARS-CoV-2 outbreak is over [5]. Risk of emerging infectious diseases is a key component of sustainable development planning, and the processes that drive disease emergence risk interact with those necessary to achieve multiple societal goals. The appearance and reappearance of such viral outbreaks can compromise the United Nations Sustainable Development Goals (SDGs), too, which are set to be reached by 2030. As consequences of environmental change, emerging infectious diseases may directly impact human health outcomes. SDGs 2 (zero hunger), 3 (good health and well-being) and 15 (life on land) are linked through the shared influence of environmental change. These interactions increase the transmission risk of infectious diseases while decreasing the disease regulation capacity, food production, and biodiversity [5]. The current lack of focus on these interactions generates policy blind spots that must be addressed to ensure that sustainable development efforts are not counterproductive and do not compromise global health security. So far, policies to deal with emerging infectious disease risk have largely remained reactive, focusing on outbreak investigation and control and development of vaccines and therapeutic drugs targeting pathogens [5]. Recently, 69 countries have been engaged in finalizing the Global Health Security Agenda (GHSA) 2024 Framework to evaluate their health security capacity through proper planning and resource mobilization in the prevention, early detection, and effective response to infectious disease threats in alignments with SDGs 2 and 3 [224].

Furthermore, the impacts of the SARS-CoV-2 pandemic is also likely to extend to other SDGs including SDGs 1 (no poverty—via decline in economic activities leading to income reduction), 4 (quality education—via closure of schools and limited internet access in some parts of the world restricting students’ access to learning), 5 (gender equality—via differential social repercussions among men and women, primarily on clinical aspects), 8 (decent work and economic growth—via shutdown of companies including small businesses in unorganized sectors thereby increasing unemployment), 10 (reduced inequalities—via worsening economic disparities), and 16 (peace, justice and strong institutions—via increased likelihood of conflicts blaming one another for the worsening situations) [225]. It is too early to understand how much the pandemic will affect the fight against poverty, however, the negative impact on poverty reduction will be substantial and swift. For the first time since 1998, annual poverty rates are expected to increase and in 2020 alone, the pandemic could increase the number of people living in extreme poverty by 88 million to 115 million (particularly those living under US $1.90-a-day). The poorest are enduring the highest incidence of the disease and suffering the highest death rates worldwide counteracting the progress made in the fight against poverty in the last 5 years. COVID-driven poverty is making inroads in populations that had been relatively spared—more urban and educated than the chronic poor, more engaged in informal services and manufacturing and less in agriculture [226]. In fact, the World Bank estimates on the impact of SARS-CoV-2 outbreak on global poverty under worsening growth and inequality are considerably larger than the increase in inequality during past pandemics (estimated to be around 1.25% five years after the pandemic), and underscores the unprecedented nature the global pandemic COVID-19 [227].

It requires responsiveness and robust healthcare systems, along with proper planning and implementation, to stop the spread of the disease. SARS-CoV-2 has emerged as one of the most highly contagious viruses of all time and is spreading at a rapid pace. It appears that the lessons learned from earlier viral epidemics were not sufficient, and this has left countries around the world ill-prepared to deal with the challenges posed by the COVID-19 pandemic [228]. In this section, we address several key aspects that may be regarded as lessons learned from the COVID-19 pandemic and compare them with earlier viral epidemics. Rigorous analysis of data and assessment of these lessons will be crucial to curbing the spread of the disease and combating future epidemics.

### 6.1. Prompt Reporting

One of the most compelling lessons learned from all earlier viral epidemics is that there is a need to report, promptly and openly, cases of any disease with a potential for international spread. This has become especially important in the closely interconnected and highly mobile world of the modern era. During the SARS-CoV epidemic, a resolution was passed by the WHO in the World Health Assembly held in 2003, where all countries were urged to report cases promptly and transparently and to provide information requested by the WHO that could help prevent the international spread [223]. This was also a problem during the MERS-CoV epidemic, in which the approach of early reporting was compromised. The International Health Regulatory Emergency Committee of WHO reported that the sharing of data on this disease was limited and fell short of expectations [229]. A robust approach to the early detection of MERS-CoV-infected patients is critical and needs to be strictly followed for the prevention of MERS-CoV [230]. During the H1N1 pandemic, country officials in Mexico underestimated incidence rates in mid-March in 2009. Although this was not a peak season for viral outbreaks, routine surveillance detected an unexpected increase in cases of influenza-like illness in mid-April 2009 [231], which was later confirmed to be the H1N1 virus. At that time, the government and public health organizations still lacked the knowledge of early detection and the importance of prompt reporting. When cases increased throughout April 2009, awareness about early detection and the implementation of effective control measures increased [232]. A similar situation arose during the Ebola outbreak in West Africa, which revealed shortcomings in the national and international capacity to detect, monitor and respond to infectious disease outbreaks. During the Ebola outbreak, the lack of early detection and effective management of the disease fueled the emergence of a public health crisis [233]. One effective solution to problems regarding case detection and estimation could be the development and deployment of rapid, point-of-care diagnostics tests linked to modern information technology [234]. In the case of HIV, the implementation of a surveillance system is limited by privacy concerns and ethical issues. The ethical issues of collecting HIV data by public health surveillance systems can be resolved by applying three well-known principles that were first advanced to protect human subjects in biomedical research: beneficence, respect for persons and justice [235]. Such prejudicial stigma is not associated with SARS-CoV-2 infection. Protecting the privacy of COVID-19 patients may become a matter of ethical dilemma, as it may also pose a potential risk to other members of society [236].

As a public health emergency of international concern, the early detection and isolation of SARS-CoV-2 patients are of paramount importance [237]. The importance of early detection and testing significantly increases for a virus such as SARS-CoV-2, which has a high replication rate and infection potential. The CDC has implemented aggressive measures to stop the spread of disease, including the identification of cases arriving from mainland China to the USA and ensuring their appropriate care [238]. European countries such as Italy, France, Germany, Spain, Austria and Switzerland have adopted some drastic steps to mitigate the spread of the virus, such as the cancellation of annual events in which gatherings of more than 1000 people occur; a ban on train travel via key international routes; the shutdown of educational institutions, restaurants and businesses; and a ban on customary greeting practices and sporting events [239]. The first case of the disease was detected in December 2019 in China, and it was not until 7 January 2020 that the causative agent of the disease was identified to be SARS-CoV-2 [240]. 

### 6.2. International Collaborations

In the wake of a pandemic, international collaborations hold immense importance, wherein scientists, clinicians and public health experts across the globe need to work for the benefit of mankind and dispense with competition in their respective fields. During the SARS-CoV epidemic, conclusive identification of the virus was declared one month after the laboratory network was established which was shortly followed by the complete genome sequencing of its RNA by the participating scientists. The network of clinical experts provided a platform for the comparison of patient management strategies to inform the world of treatments and strategies that were effective. Long-term international collaborations helped us to understand the mode of transmission of SARS-CoV and the clinical spectrum of the disease [223].

During the emergence of MERS-CoV, a rapid increase in research activity on the disease was observed from 2012 to 2015. During this period, a total of 883 research papers were published in different languages, with the USA being the highest contributor, followed by the Kingdom of Saudi Arabia [194]. Collaborative research demonstrated the prioritization of the search for an appropriate vaccine as well as effective medications for the treatment of MERS-CoV [241].

In response to the H1N1 pandemic in 2009, the WHO put a large amount of work into global prevention and control efforts and also adjusted prevention and control strategy priorities to fall in line with the global influenza outbreak, to which countries worldwide were also proactive in their response. Ninety-eight institutions across 73 countries were able to perform polymerase chain reaction (PCR) tests to detect H1N1 in humans, which worked as a necessary monitoring system of new confirmed cases worldwide [242]. International collaboration and solidarity were also necessary for vaccine development, and various institutions across the world worked collectively in response to the global pandemic. The WHO urged pharmaceutical manufacturers to prepare vaccines at full capacity to ensure fair distribution among all countries. All of these global efforts led to the end of the H1N1 pandemic in 2010 [243]. 

During the Ebola outbreak, no infrastructure to conduct clinical trials was available in the affected countries before the outbreak, and the lack of coordination fostered competition among teams over trial locations and trial participation [244]. It is unrealistic to assume that all necessary planning and coordination activities can be efficiently conducted after an epidemic begins or while it is ongoing. Hence, research must begin much more quickly during the inter-epidemic period in order to increase the likelihood of successful international collaborations during such epidemics. Contrary to the therapeutic trial, the clinical trials of the Ebola vaccine were supported by improved coordination among international stakeholders, researchers and regulators [244].

In the case of the HIV epidemic, global efforts were also necessary to control the spread of infection. The goal was to improve HIV prevention research through scientific integration. Both local and international efforts were required to identify research gaps and to discuss promising prevention strategies [245]. To this end, activities and outcomes such as facilitating knowledge transfer and exchange, establishing and strengthening multi-stakeholder partnerships, and identifying new and emerging priorities in this field were necessary, for which an international community-academic-clinical research collaborative using a community-engaged approach was envisaged [246].

The solution to the current SARS-CoV-2 pandemic is primarily based on the knowledge derived through in-depth research. Global efforts are now focused on understanding the properties and etiopathology of SARS-CoV-2 to develop interventions, including vaccines and specific treatments. Furthermore, researchers that have adequate skill sets with appropriate funds are also focused on finding ways to more effectively convey information to the general public so as to avoid panic in the face of uncertainty [247]. Widespread scientific collaborations in multidisciplinary fields are needed to establish practical methods for large-scale disinfection treatment to inactivate SARS-CoV-2 in different environmental conditions, which must be accompanied by effective research in vaccine development [248]. In recent weeks, doctors, researchers, engineers and scientists from all fields of knowledge have shown an unprecedented spirit of collaboration to confront the COVID-19 pandemic [249]. To better understand the dynamics of specific infections, researchers are assisted by mathematical and epidemiological simulation models in anticipating and controlling future epidemics [250]. Two mathematical models that are being used by public health agencies for the current COVID-19 pandemic are the stochastic model and susceptible–infected–recovered model [251,252]. In such an emergency context, universities in engineering and materials sciences work synergistically with multiple companies by performing validation tests for newly made PPE kits and producing ventilators and respirators [253]. The openness of data sharing and collaborative work is important for developing countries that lack comprehensive research structures. To prepare for future epidemics, new technologies must be developed that can drive research progress, and well-trained and funded collaborations across research disciplines must be enabled. Most importantly, research programs must be established in the inter-epidemic period so that we can prepare ourselves before the epidemic arrives [248].

### 6.3. Strengthening of Healthcare Facilities

A strong healthcare system is the most vital weapon of a country in its fight to control an epidemic. Thus, the top priority of a country remains the strengthening of its healthcare facilities during the inter-epidemic period. The SARS-CoV epidemic exposed weakened health facilities with many longstanding and intractable problems, such as requirements for isolation wards, long periods of intensive care, mass screening, contact tracing, active surveillance and quarantine facilities [223]. After the outbreak, some of the traditional and seemingly intractable problems in healthcare systems were corrected in fundamental ways in China, the lessons from which will help in shaping the capacity of healthcare facilities elsewhere and in future epidemics [223]. The H1N1 pandemic substantially impacted healthcare systems of the world, particularly through an increased burden on the emergency departments of hospitals. In the USA alone, hospitals experienced a doubling in the demand for emergency services due to influenza. Healthcare facilities experienced a high surge in inpatient admissions, and increased mortality from selected clinical conditions was associated with both pre-pandemic outcomes and the patient surge, which also highlighted the linkage between daily hospital operations and disaster preparedness [254]. During the emergence of the Ebola epidemic, the need for a robust healthcare system was particularly apparent, especially in the developing nations of Africa. The most profound consequences of poor healthcare facilities were felt in West Africa due to the failure of health governance [255]. Poor organization, lack of prompt political decision making and subsequent inadequate government response in many African countries were among the reasons behind the failure to control the Ebola epidemic. The crisis fueled the lack of faith of citizens in governments, which further aggravated social tension [256]. The prolonged civil war in Liberia and chronic political instability in Guinea exacerbated the Ebola situation [257]. Additionally, the global response mechanisms were relatively deficient as a result of poor infrastructure, fragmented health systems and inadequate experimental treatments. A number of discrete areas of systemic weakness were identified in the Ebola-struck African regions, which required immediate attention: training of healthcare professionals, poor infection surveillance and response systems, infection control, contact tracing, laboratory systems, networking and coordination systems and community engagements [258]. Similar situations also arose during the HIV outbreak, largely in African countries where infrastructure was poor, which hindered healthcare workers from effectively performing their duties. The AIDS epidemic added to the burden of diseases, and as a result, demands for proper healthcare facilities increased to a large extent in affected countries [259]. To effectively combat such epidemics, healthcare facilities in poorer countries must be improved, for which developed countries and international organizations must come forward.

To date, COVID-19 is the largest pandemic of the century and has resulted in unprecedented global health crises. This has challenged the healthcare facilities of affected countries and stretched their limits to a considerable extent. Hence, an efficient healthcare system is of utmost necessity in order to respond to a pandemic of such magnitude. Lower- and lower-middle-income countries are experiencing major challenges in handling this pandemic because of pre-existing shortcomings in public health infrastructure, combined with the demand in the care and management of SARS-CoV-2 patients [260]. The most affected developing countries are finding it difficult to fairly and equally provide healthcare facilities amidst rapidly increasing cases on a daily basis. Even developed countries are facing trouble in this regard as a result of rapid increases in confirmed cases. Since its first reported case in February 2020, COVID-19 cases in the US have risen dramatically, and government and healthcare officials are experiencing hardships inequitably providing healthcare facilities to all infected patients. Furthermore, unemployment has risen slightly, which has resulted in financial constraints for the population. About 70% of US citizens have stated that the economy is in a state of depression due to the COVID-19 pandemic [261]. In the UK, the government is facing persistent shortages of PPE for health workers, and the majority of them have to rely on donations [262]. Furthermore, the UK has experienced the worst economic recession this year, with about 14% shrinkage in the economy; thus, the government eased the lockdown to provide a boost to the economy [263]. There is a continued increase in the demand for hospital beds, safety kits, N95 masks and ventilation facilities in most of the affected countries. With a projected second wave of infection and no convincing confirmation of any effective vaccine, emphasis should be placed on effective planning, communication and coordination between centralized health policymakers and health managers who work in primary care settings to ensure overall preparedness, both now and for future pandemics [264].

### 6.4. Interventions

During the SARS and MERS-CoV outbreaks, standard public health intervention strategies were followed; these include treating patients with antiviral drugs, following social distancing norms and implementing quarantine measures. By effectively applying these measures, the burden on healthcare facilities may be reduced until a vaccine is developed [223]. During the H1N1 pandemic, both pharmaceutical and social distancing interventions were recommended by the WHO and other countries. It has been reported that the aggressive use of antiviral drugs, together with extended school closures, may substantially slow the rate of influenza transmission. Furthermore, computer modeling and simulations were used during the early stages of the pandemic to determine the potential effectiveness of social distancing and antiviral drug therapy interventions [265]. Intervention strategies adopted during the Ebola outbreak delivered positive results by reducing the spread of the epidemic. Interventions were organized around five major strategies: the building of Ebola treatment units to safely isolate and treat infected individuals, the setting up of laboratories to test and identify those infected, identification of positive cases through surveillance and contact tracing, safe burial and body management, and social mobilization to educate people about the spread of Ebola. No single intervention stopped the epidemic; rather, the combined actions acted as the driving force behind containing the spread of the epidemic, while some interventions were less likely than others to have significant effects [266]. Combinations of various intervention strategies were used during the HIV outbreak in the absence of a vaccine. These interventions integrated efficacious behavior and biomedical strategies to offer potential strategies to reduce new HIV infections. Prevention strategies other than vaccination showed promising results and included the use of microbicides applied either to the vagina or to the rectum, pre- and post-exposure prophylaxis along with the use of anti-retroviral medication, medical male circumcision, HIV testing, linkage and retention in HIV care and enhanced anti-retroviral adherence among HIV seropositive individuals. In addition to these measures, there will be an immediate and urgent need for effective strategies to integrate and evaluate the combination of HIV prevention interventions in the future [267]. Beyond the aforementioned intervention lessons learned from past pandemics, the current management of the SARS-CoV-2 pandemic focuses primarily on the ‘flattening the curve’ approach. The aim of this strategy is to slow the spread of an epidemic caused by an infection so that the peak number of individuals who require health support does not exceed its capacity, and the healthcare system can work efficiently without experiencing excessive constraints [268]. These techniques are adopted once efforts to contain an outbreak have failed and an effective vaccine is yet to be developed. Although the first COVID-19 vaccine ‘Sputnik V’ has already been registered, non-vaccine intervention procedures still need to be followed until the commencement of large-scale immunization programs. At present, medical interventions include supportive therapies, with no specific treatment procedure. Medications such as remdesivir (an anti-retroviral) and dexamethasone (a corticosteroid) are being tested, with a mixed response rate [269]. SARS-CoV-2-induced animal models have also confirmed the inhibition of viral replication by remdesivir: promising results include the reduction of viral load, alleviation of mild symptoms and improvement in pulmonary lesions [270]. Although remdesivir has been shown to inhibit SARS-CoV-2 both in vitro and in vivo [271], randomized trials conducted to date have been unable to demonstrate any difference in mortality [272,273]. One of these trials reported a faster time to recovery as compared with the control [271]. Forthcoming confirmatory trials may reveal its efficacy as well as safety [273]. Dexamethasone has also been found to possess anti-inflammatory and immunosuppressant effects. It has shown positive results in critically ill COVID-19 patients, which includes a reduction in mortality by about one-third in ventilated patients [274]. Patients with critical symptoms are kept on ventilation. Other public health interventions include social distancing; effective distribution of PPE, such as masks, gowns and gloves; rotating shifts of healthcare providers to limit exposure and allow recuperation; and identification of infected cases and those with travel history, followed by their isolation in appropriate separate screening locations and quarantine centers. All of these measures may reduce the burden on healthcare facilities and prevent the spread of disease [275].

## 7. Perspective

The emergence of deadly viruses and their global outbreaks pose threats to the world’s public health and economy. The COVID-19 pandemic is proving to be an unprecedented disaster, especially in terms of the health, social and economic aspects. Both high- and low-income countries are facing catastrophic consequences [276]. This is the third time that a virus of the family Coronaviridae has caused an epidemic of such a massive scale in the 21st century. In this scenario, the development of new drugs and clinical trials of existing drugs are priorities, along with the design and development of vaccines for such viruses. Additionally, the natural animal reservoirs of these viruses should be identified, and restrictions should be imposed on their consumption. Lessons from SARS-CoV and MERS-CoV suggest that focus should be placed on establishing animal models that can reproduce and mimic various aspects of the human disease so that further research can be conducted on the development of a vaccine [277]. The global approach is to isolate the world population and to stop the spread of the disease while the vaccine is being developed. Certain challenges lie in the development and testing of vaccines to rapidly control SARS-CoV-2, which requires international collaboration [278]. With the proper implementation of prevention measures, a lower incidence of COVID-19 and other hygiene-linked diseases can be achieved. This review aims to highlight the lessons learned from the epidemics of this century, especially COVID-19; however, only after this pandemic ends will one be able to assess the actual health, social and economic impacts of the disease and a complete picture will emerge to guide the response to future pandemics [279].

## Figures and Tables

**Table 1:** Comparison of the characteristics, pathophysiologies and management of viral pandemics.

	SARS-CoV-2	SARS-CoV	MERS-CoV	Ebola	H1N1	HIV	References
**Outbreak year and location of first reported cases**	2019 (Wuhan, China)	2003 (Southern China)	2012 (Saudi Arabia)	1976 onwards (Central Africa)	2009 (North America)	1981 onwards (West Central Africa)	[172]
**Outbreak countries**	More than 215 countries, including the USA, India, Brazil, China, Japan, Korea, Italy, etc.	29 countries, including China, Vietnam, Singapore and Canada	More than 27 countries, mainly in Saudi Arabia, South Korea, Jordan and Qatar	Africa, the Americas, South East Asia, Europe, Eastern Mediterranean, Western Pacific	Africa, Europe, the Americas, South-East Asia	More than 130 countries, including the USA, China, India, etc.	[173,174]
**Natural reservoir**	Not identified	Bat	Bat	Fruit bats, porcupines and non-human primates	Human, avian, swine	Chimpanzee	[173,175]
**Receptor**	ACE2, TMPRSS2, sialic acid	ACE2, CD206, sialic acid	DPP4 (CD26), sialic acid	TIM1 (NPC1)	sialic acid	CD4	[79,80,81,176,177,178]
**Case Fatality Rate**	Not identified, at least 2–3%	10%	34.4–37%	50–63%	0.02–0.4%	80–90%	[179,180]
**Hospitalization rate**	~19%	Most cases	Most cases	25–90%	16.19–58.76%	>34.2%	[181,182,183]
**Community attack rate**	30–40%	10–60%	4–13%	5–30%	10–20%	23%	[184]
**Basic reproductive number (R_0_)**	1.4–6.4	2–5	<1	1.9	1.3	2–5	[179,185]
**Median incubation time**	5.2 days	5 days	5 days	2-21 days	1–7 days	5–70	[186,187]
**Clinical symptoms**	Fever (98%), cough (77%), dyspnea (63.5%), myalgia (11.5%), malaise (35%) and so on	Fever (>99%), cough (62%–100%), chills or rigor (15%–73%), diarrhea 20%, dyspnea (40%)	Fever (77%), cough (90%), dyspnea (68%), sputum production (40%), odynophagia (39%), digestive system/signs (20%), hemoptysis (4.3%), myalgia (43%) and headache (20%)	Fever, fatigue, muscle, pain, headache, sore throat, vomiting, diarrhea, rash, kidney and liver impairments and, in some cases, internal and external bleeding (e.g., oozing from the gums, blood in the stools). Laboratory findings include low WBCs and platelet counts and elevated liver enzymes.	Fever, chills, cough, sore throat, runny or stuffy nose, red eyes, body aches, headache, fatigue, diarrhea, nausea and vomiting	Muscle aches (85%), fatigue (84%), bloating (82%), fever (79%), headache (73%), memory loss (73%), cough (74%), poor appetite (74%), diarrhea (71%) and nausea (72%)	[50,182,188,189,190]
**Radiology**	Critically ill with bilateral multiple lobular and subsegmental areas of consolidation; mild ill with bilateral ground-glass opacity and subsegment alareas of consolidation, almost 100% of patients with abnormal CT	Unilateral/bilateral ground-glass opacities or focal unilateral/bilateral consolidation. The rate of abnormal chest radiography or CT was >94%	Unilateral/bilateral patchy densities or infiltrates, bilateral hilar infiltration, segmented/lobar opacities, ground-glass opacities and possible small pleural effusions. The rate of abnormal chest radiography or CT was between 90% and100%	Aerosolized virus would be unlikely to produce discrete, radiographically visible, pulmonary lesions.	Initial chest radiographs show central or peripheral pulmonary GGO and consolidations with patchy or nodular appearance; multizonal and bilateral peripheral opacities are associated with adverse prognosis.	Bronchiectasis, with ill-defined centrilobular micronodularity and branching structures to mucous impaction in the bronchioles, along with cavitation.	[186,191,192]
**Cytokines**	Increased levels of IL-1β, IL1RA, IL-7, IL-8, IL-9, IL-10, basic FGF, GCSF, GMCSF, IFN-Ɣ, IP10, MCP1, MIP1α, MIP1β, PDGF, TNF-α and VEGF; Critically ill patients have high levels of GCSF, TNF-α and Th2 cytokines (e.g., IL-4 and IL 10)	Increased levels of IL-1β, IL-6, IL-12, IFN-Ɣ, IP10 and MCP-1	Increased concentrations of proinflammatory cytokines (IFN-Ɣ, TNF-α, IL-15 and IL-17)	TNF-α, IFN-γ, IL-1RA, IL-6, IL-15, MIG, MIF, MIP-1α, MIP-1β, MCP-1, IP-10, ITAC, eotaxin, IL-2, IL-1β, IL-8, HGF, VEGF, GM-CSF and G-CSF	IL1RA, IL-6, TNF-α, IL-8, MCP-1, MIP1β and interferon-inducing protein-10	Increased levels of TNF-α, TNF-β, IFN-Ɣ, IL-1, IL-2, IL-6, IL-7, IL-10, IL-13, IL-15 and IL-16	[148,186,193,194,195]
**Treatment**	Corticosteroids, remdesivir, combination of lopinavir and ritonavir, type I interferon and so on	Lopinavir and ritonavir, corticosteroids, IFN-Ɣ, IVIG	IFN-Ɣ, lopinavir and ritonavir, mycophenolic acid	During the 2018 eastern Democratic Republic of the Congo outbreak, two out of four investigational treatments initially available to treat patients with confirmed Ebola, are still in use. These two are REGN-EB3 and mAb114. In addition, treatments include fluid intake and intravenous electrolytes, oxygen therapy and using medication to manage blood pressure, vomiting, diarrhea, fever and pain.	Oseltamivir (Tamiflu),peramivir (Rapivab) andzanamivir (Relenza) appeartowork best, although some types of swine flu do not respond to oseltamivir.	Anti-retroviral therapy, which includes medications such as abacavir, efavirenz, enfuvirtide, atazanavir, maraviroc, dolutegavir, ibalizumab, cobicistat, etc.	[180,196,197,198]

ACE2: Angiotensin-converting enzyme 2; CD: Cluster of differentiation; CT: Computed tomography; DPP4: Dipeptidyl peptidase IV; FGF: Fibroblast growth factor; G-CSF: Granulocyte colony-stimulating factor; GGO: Ground-glass opacities; GM-CSF: Granulocyte/monocyte colony-stimulating factor; H1N1: Influenza virus A subtype H1N1; HGF: Hepatocyte growth factor; HIV: Human immunodeficiency virus; ICU: Intensive care unit; IFN: Interferon; IL: Interleukin; IL1RA: Interleukin-1 receptor antagonist; IP: Interferon γ-induced protein; ITAC: Interferon-inducible T-cell α chemoattractant; IVIG: Intravenous immunoglobulin; mAb114: Monoclonal antibody 114; MCP: Monocyte chemoattractant protein; MERS-CoV: Middle-East respiratory syndrome coronavirus; MIF: Macrophage migration inhibitory factor; MIG: Monokine induced by gamma; MIP: Macrophage inflammatory protein; NPC1: Neimann-Pick C1 protein; PDGF: Platelet-derived growth factor; REGN-EB3: cocktail of three monoclonal antibodies REGN3470, 3471 and 3479 developed by Regeneron Pharmaceuticals; SARS-CoV: Severe acute respiratory syndrome coronavirus; SARS-CoV-2: Severe acute respiratory syndrome coronavirus 2; Th2: T-helper cell type-2; TIM1: T-cell immunoglobulin and mucin domain 1; TMPRSS2: Transmembrane serine protease 2; TNF: Tumour necrosis factor; VEGF: Vascular endothelial growth factor; WBC: white blood cell.

**Table 2:** The most promising Covid-19 vaccines currently under clinical trials.

Name of Vaccine	Developer Country	Institute and Company	Mode of Action	Results to Date	References
Sputnik V	Russia	The Gamaleya National Center for Epidemiology and Microbiology	A viral vector vaccine that uses a weakened version of the common cold-causing adenovirus to introduce the SARS-CoV-2 spike protein to the body.	Researchers claim that the vaccine can induce strong antibody and cellular immune responses. However, published data on the clinical trials are not available yet.	[210,213,214,215,216,217,218,219,220,221,222]
mRNA-1273	USA	Moderna Therapeutics	An mRNA-based vaccine that mimics the coronavirus, thus training the immune system to recognize its presence.	Phase III clinical trials. Trials involving high risk and elderly showed that it is nearly 95% effective.	
BNT162b2	USA	Pfizer and BioNTech (Germany)	A nucleoside-modified mRNA that encodes an optimized SARS-CoV-2 full-length spike protein antigen. It contains a piece of the spike protein that elicits an antibody response.	Patients demonstrated a favorable overall tolerability during phase I/II trials and induction of a favorable viral-specific CD4+ and CD8+ T-cell response. Received temporary authorization from the UK Medicines and Healthcare products Regulatory Agency on 2 December, 2020. On 8 December 2020 at 6.31 am local time in London, UK, 334 days after the first reported Covid-19 death in China, Margaret Keenan, 90, became the first person in the world to receive a clinically approved vaccine.	
AZD1222	UK	The University of Oxford; AstraZeneca; IQVIA; Serum Institute of India	A non-replicating viral vector with the viral spike protein, which induces an immune response.	Currently undergoing phase III clinical trials. Phase III interim results, based on 131 cases, as declared via press release (23 November 2020) suggest that it can be up to 90% effective when a half dose is given, followed by a full dose one month later.	
Covaxin	India	Bharat Biotech; National Institute of Virology	An inactivated vaccine to trigger specific T-lymphocytes and neutralizing antibodies by the host’s immune system.	Currently undergoing phase III clinical trials. The participants of the clinical trials are reportedly healthy, adeno adverse impacts of the vaccine have been found to date.	
CoronaVac	China	Sinovac and Butanan (Brazil)	An inactivated vaccine that initiates an immune response without producing the disease.	Currently undergoing phase III clinical trials. Subjects in the phase II human trial produced antibodies with no severe adverse reactions.	
No name announced	China	Sinopharm and Wuhan Institute of Biological Products	An inactivated vaccine that is renderednon-infectious but retains enough surface proteins to set off an immune response.	Undergoing phase III clinical trials. Earlier trial phases have shown that the vaccine can trigger an antibody response with no serious adverse effects.	
JNJ-78436735	USA	Johnson and Johnson	Non-replicating viral vector. Optimal Ad26 vector-based vaccine for SARS-CoV-2	Currently undergoing phase III clinical trials. Initial data demonstrated that a single shot of the vaccine provided protection against SARS-CoV-2 in non-human primates.	
Ad5-nCoV	China	Cansino Biologics	A viral vector vaccine made using a weakened version of the adenovirus (with faulty replication mechanism) as a vehicle for introducing the SARS-CoV-2 spike protein to the body.	Currently undergoing phase III clinical trials. Phase II trials showed that the vaccine produces significant immune responses in the majority of recipients after a single immunization.	
NVX-CoV2373	USA	Novavax	It is a protein subunit vaccine made with full-length recombinant SARS-CoV-2 glycoprotein nanoparticles, adjuvated with Matrix M, which enhances immune response and stimulates high levels of neutralizing antibodies by increasing the rate of antigen-presentation in the local lymph nodes.	Currently under phase III clinical trials, this vaccine candidates demonstrated efficient binding with receptors targeted by the virus, which is a critical aspect of effective vaccine action.	
ZF2001	China	Anhui Zhifei Longcom Biopharmaceutical and Institute of Microbiology, Chinese Academy of Sciences	It is an adjuvated recombinant protein subunit vaccine expressed in CHO cells. It probably elicits protective action against the virus by increasing the level of neutralizing antibody and IgG antibody.	Currently under phase III clinical trials, the vaccine candidate showed promising results during the earlier phases by generating immune response.	

Ad26: Adenovirus type 26; CD: Cluster of differentiation; CHO: Chinese hamster ovary; SARS-CoV-2: Severe acute respiratory syndrome coronavirus 2.
